# Supplementary figures and images for: Cortical softening elicits zygotic contractility during mouse preimplantation development
Source: PLoS Biol. 2022 Mar 24;20(3):e3001593. doi: 10.1371/journal.pbio.3001593 (PMC8982894; doi:10.1371/journal.pbio.3001593)

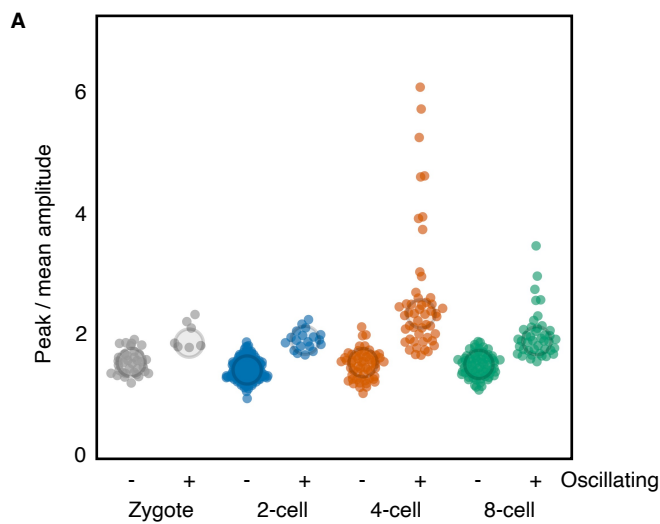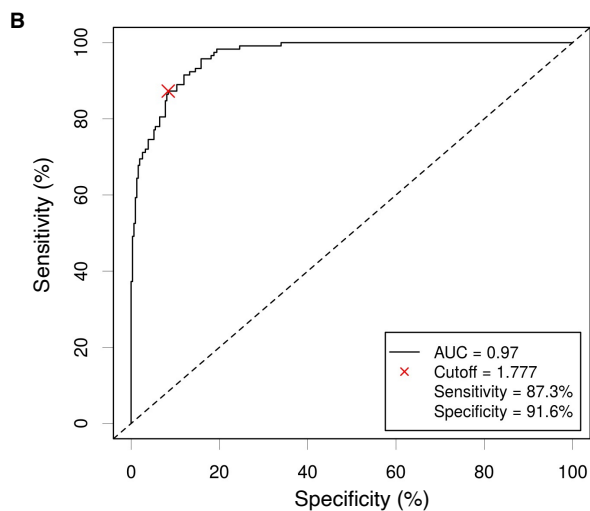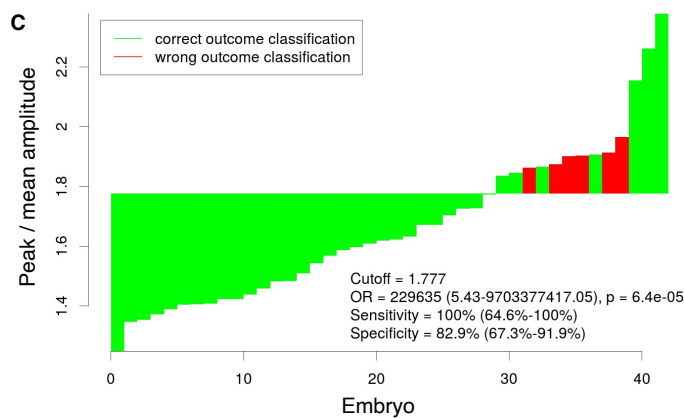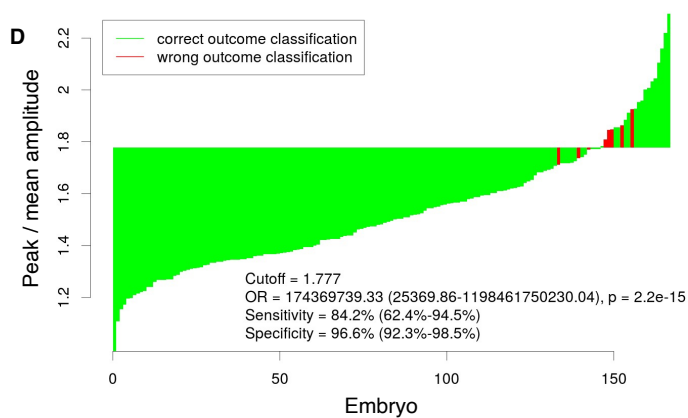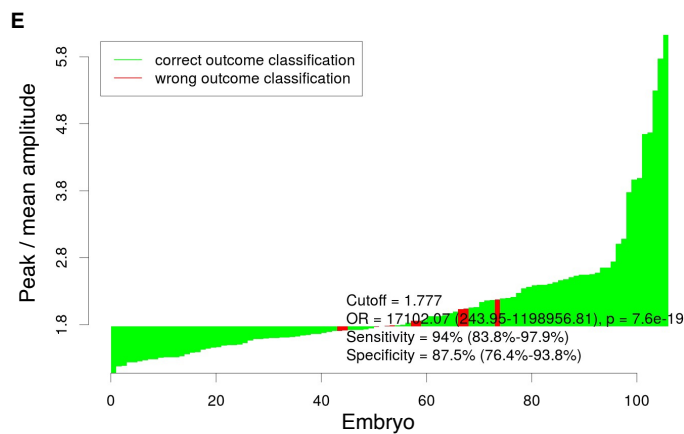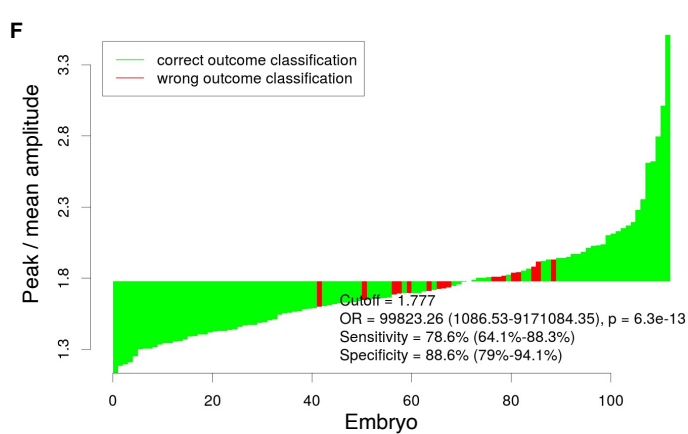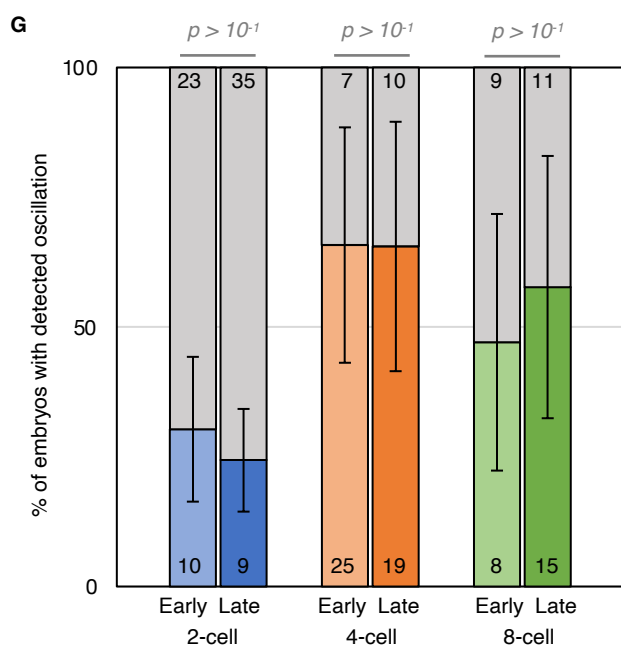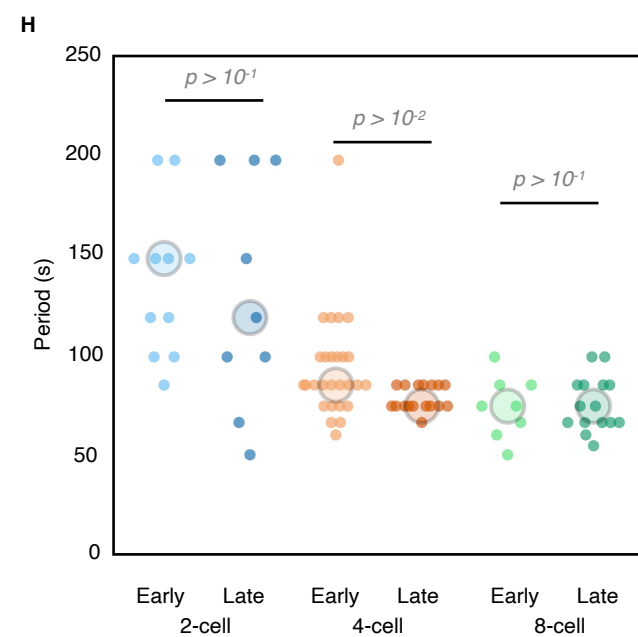

Supplement: S1 Fig — Analysis of PeCoWaCo during cleavage stages using PIV and Fourier transform. (A) Peak value divided by mean signal of the respective power spectra after Fourier analysis of PIV data for embryos at the zygote, 2-, 4-, and 8-cell stages. Embryos are classified as showing a visible oscillation (+) or not (−), as assessed visually. (B) ROC curve resulting from CutOffFinder [62] showing that a threshold of 1.777 for the peak/mean amplitude (red cross) yields a maximized AUC of 0.97. (C–F) Classification of zygote (C), 2- (D), 4- (E), and 8-cell stages (F) using a threshold of 1.777 for the peak/mean amplitude. Red indicates a mismatch between the visual assessment and the use of the threshold to determine whether a given embryo oscillates or not at a given time point. (G) Proportion of early and late 2-cell (blue, n = 33 and 44), 4-cell (orange, n = 38 and 29), and 8-cell stage (green, n = 17 and 26) embryos showing detectable oscillations after Fourier transform of PIV analysis. Error bars show SEM. Chi-squared p-values comparing different stages are indicated (S2 Table, S1 Data). Light gray shows nonoscillating embryos. (H) Oscillation period of early and late 2-cell (blue, n = 11 and 9), 4-cell (orange, n = 27 and 17), and 8-cell (green, n = 8 and 15) stages embryos. Larger circles show median values. Student t test p-values are indicated (S2 Table, S1 Data). AUC, area under the curve; PeCoWaCo, periodic cortical waves of contraction; PIV, Particle Image Velocimetry; ROC, receiver operating characteristic. (PDF) [file pbio.3001593.s001.pdf]

**A**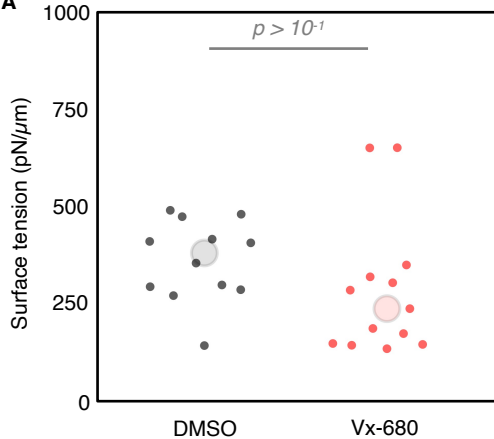**B**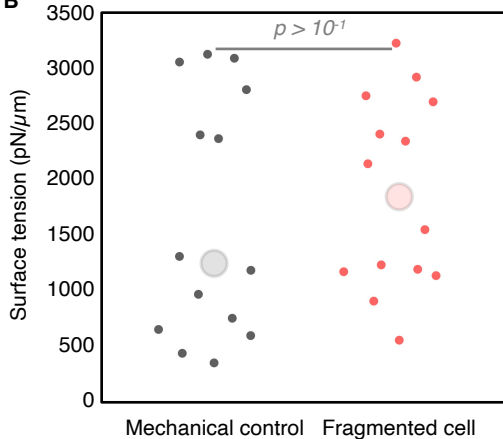

Supplement: S2 Fig — Surface tension of Vx-680 and fragmented embryos. (A) Surface tension of embryos treated with DMSO (n = 12) or Vx-680 (n = 13). Student t test p-value is indicated (S4 Table). Larger circles show median values. (B) Surface tension of mechanical control (n = 14) or fragmented cells (n = 14). Student t test p-value is indicated (S4 Table). Larger circles show median values. (PDF) [file pbio.3001593.s002.pdf]

**A**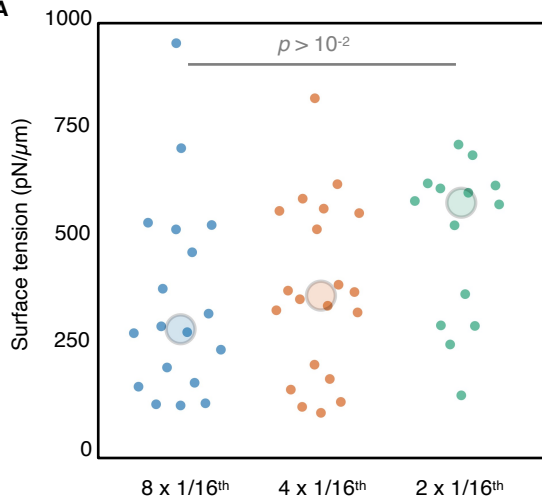**B**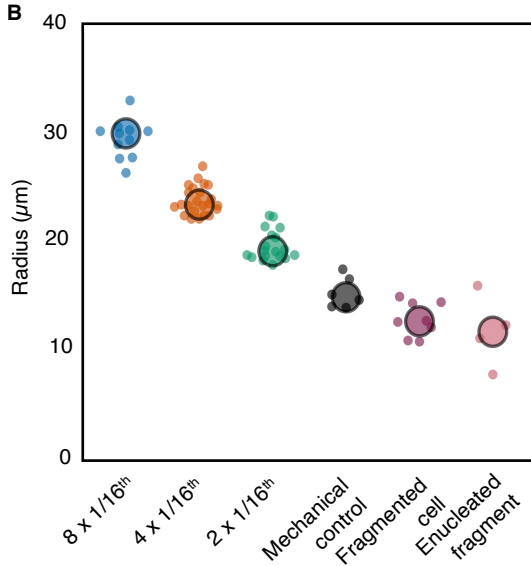

Supplement: S3 Fig — Surface tension of fused blastomeres. (A) Surface tension of cells resulting from the fusion of 8, 4, or 2 16-cell stage blastomeres (n = 18, 20 and 14 embryos, respectively). Student t test p-value is indicated (S6 Table, S1 Data). Larger circles show median values. (B) Radius of fused and fragmented 16-cell stage blastomeres. Larger circles show median values (S1 Data). (PDF) [file pbio.3001593.s003.pdf]

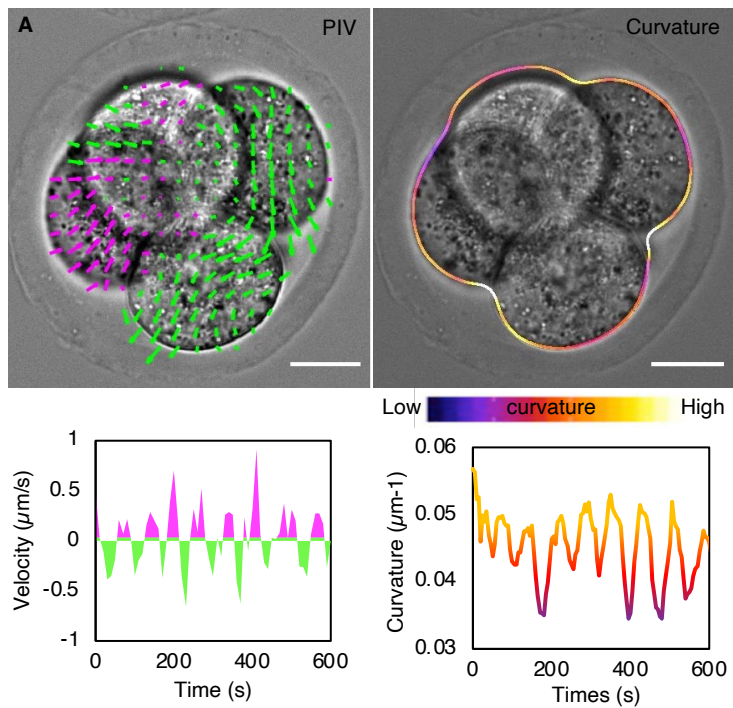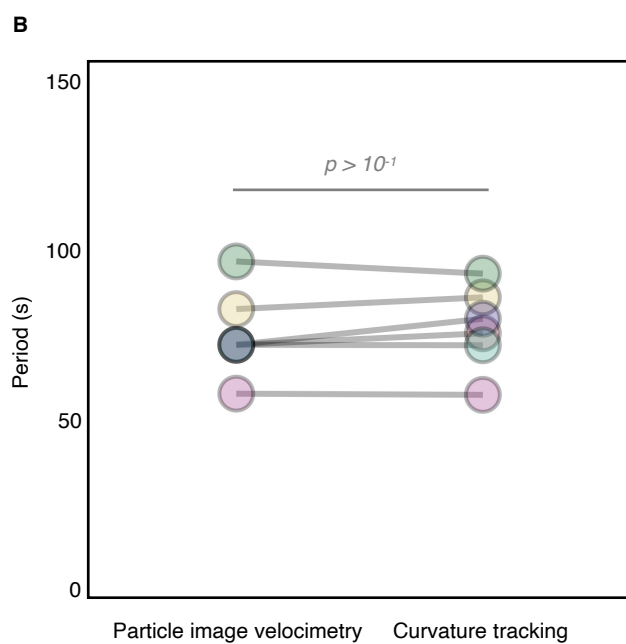

Supplement: S4 Fig — Comparison of PIV and curvature analyses of PeCoWaCo. (A) Representative images of a 4-cell stage embryo overlaid with a subset of velocity vectors from PIV analysis (top left) or a color coded local curvature analysis (top right). Velocity over time for a representative velocity vector (bottom left) or local curvature measurement (bottom right) for the bottom most blastomere. White scale bar, 10 μm. Colored scale bar indicates curvature. See S7 Movie. (B) Oscillation period of 4-cell stage embryos (n = 6) as determined by PIV or curvature tracking analyses. Pairwise Student t test p-value is indicated (S1 Data). PeCoWaCo, periodic cortical waves of contraction; PIV, Particle Image Velocimetry. (PDF) [file pbio.3001593.s004.pdf]

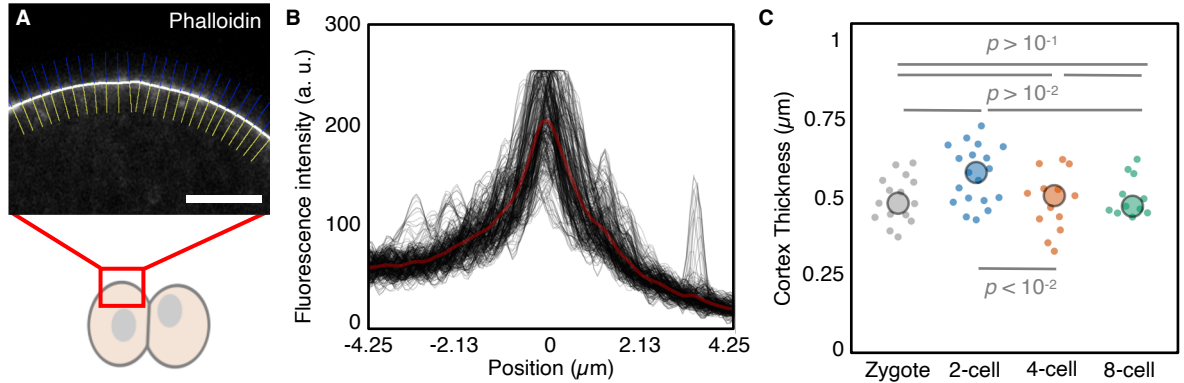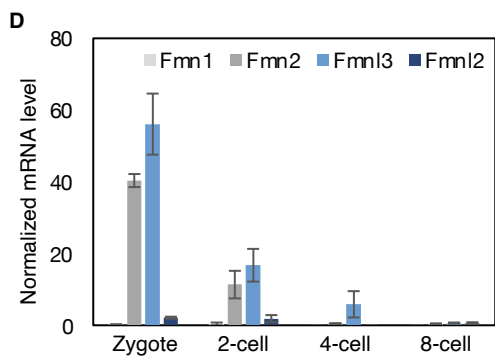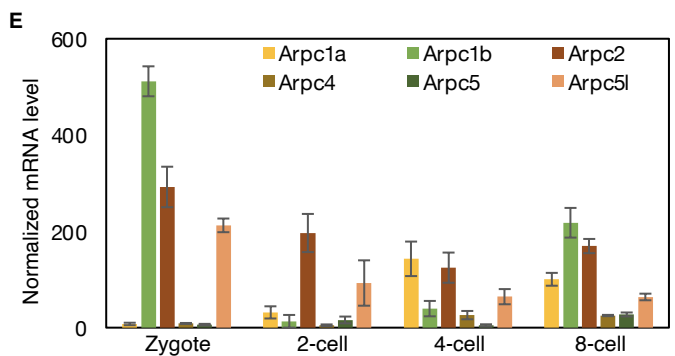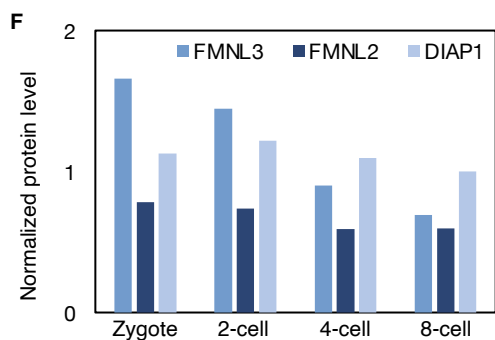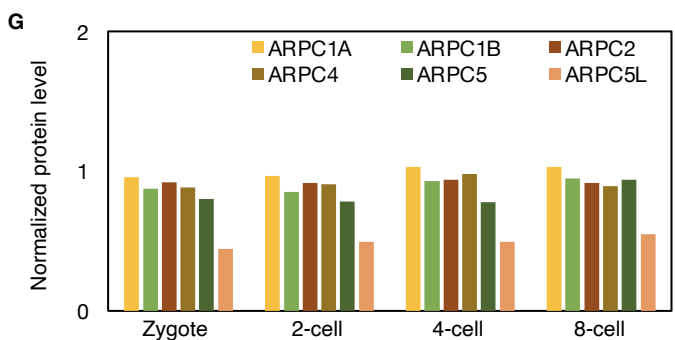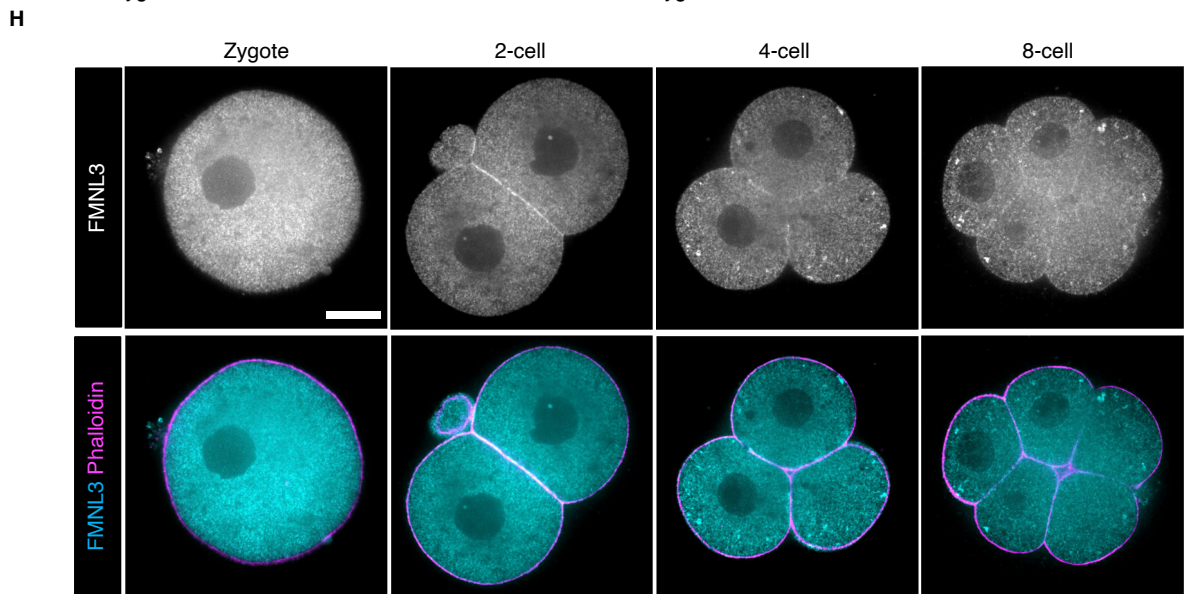

Supplement: S5 Fig — Molecular and structural characterization of the actomyosin cortex during cleavage stages. (A) Representative image of the actin cortex of a Phalloidin-stained 2-cell stage embryo acquired using super resolution microscopy. Scale bar, 5 μm. Overlaid white line shows the segmentation of the cortex, and orthogonal blue and yellow lines show line scans outside and inside the cell, respectively. Schematic diagram below shows example of where in the cell such image is taken. (B) Phalloidin intensity along lines orthogonal to the cell cortex of the cell shown in (A) (black, n = 348). Mean intensity profile shown in red. (C) Cortex thickness measured at the zygote, 2-, 4-, and 8-cell stage (n = 15, 19, 13, and 11, respectively). Student t test p-values are indicated (S8 Table, S1 Data). Larger circles show median values. (D–G) mRNA (D and E) and protein (F and G) levels during cleavage stages for formins (D and F) and arps (E and G) adapted from [44]. (H) Immunostaining of FMNL3 (gray and cyan in merged image) on representative zygote, 2-, 4-, and 8-cell stage embryos. Phalloidin shown in magenta on the merged image. Scale bar, 20 μm. (PDF) [file pbio.3001593.s005.pdf]

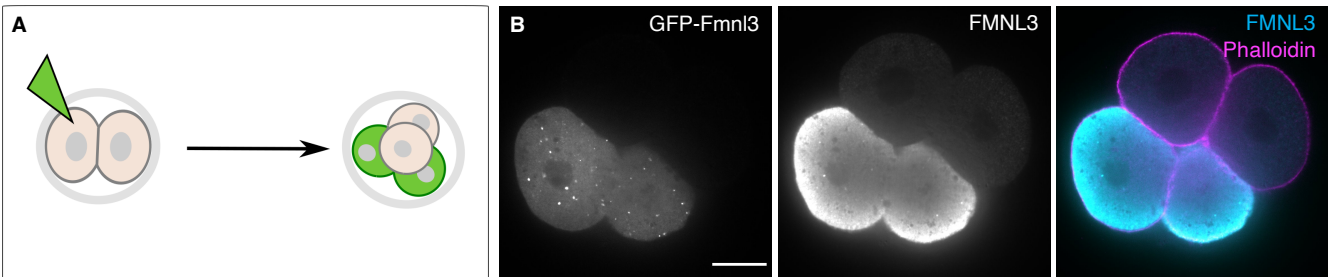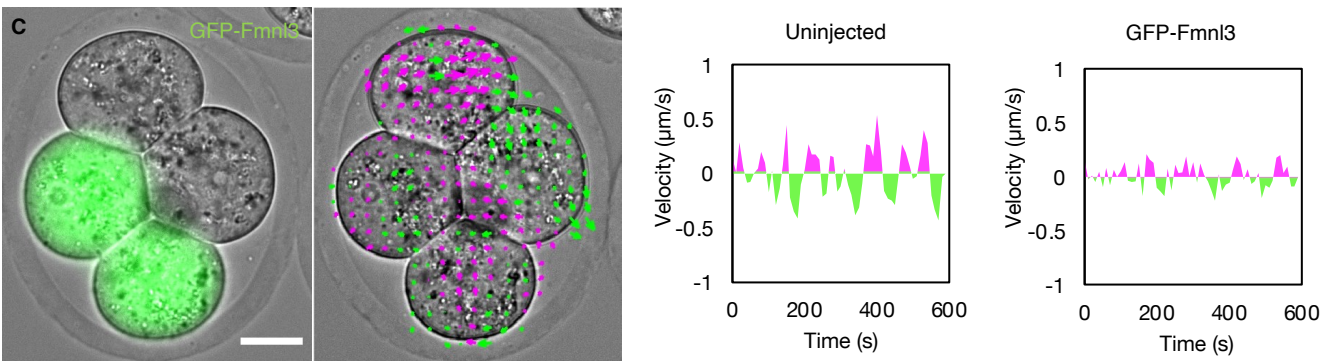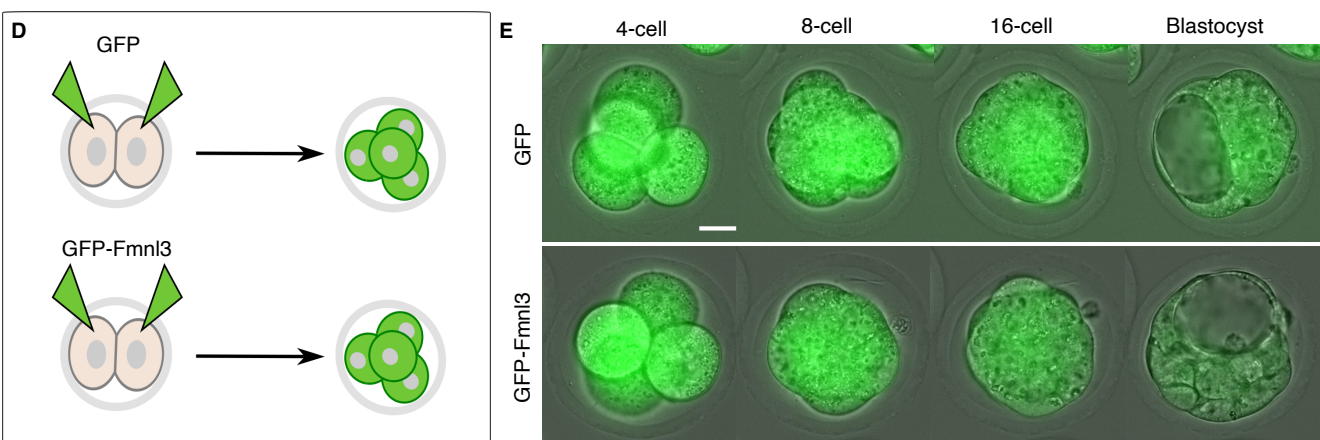

Supplement: S6 Fig — Molecular and structural characterization of the actomyosin cortex during cleavage stages. (A) Schematic diagram of GFP-Fmnl3 mRNA injection in one blastomere of a 2-cell stage embryo. (B) Immunostaining of a 4-cell stage embryo injected with GFP-Fmnl3 at the 2-cell stage. GFP-Fmnl3 is shown on the left, and FMNL3 immunostaining is shown in gray in the middle and in cyan on the right together with Phalloidin in magenta. Scale bar, 20 μm. (C) Live imaging of a 4-cell stage embryo injected with GFP-Fmnl3 at the 2-cell stage. Left shows a merged image of phase contrast and GFP-Fmnl3. Right image shows the same embryo overlaid with a subset of velocity vectors from PIV analysis. Graphs on the right are velocity over time for a representative velocity vector of an uninjected (left) and GFP-Fmnl3 injected blastomere (right). Scale bar, 20 μm. See S9 Movie. (D) Schematic diagram of GFP or GFP-Fmnl3 mRNA injection in both blastomeres of a 2-cell stage embryo. (E) Representative images of the preimplantation development of GFP or GFP-Fmnl3 expressing embryos shown at the 4-, 8-, 16-cell, and blastocyst stages. Scale bar, 20 μm. S11 Movie. PIV, Particle Image Velocimetry. (PDF) [file pbio.3001593.s006.pdf]
