# Supplementary material for: Cortical softening elicits zygotic contractility during mouse preimplantation development
Source: PLoS Biol. 2022 Mar 24;20(3):e3001593. doi: 10.1371/journal.pbio.3001593 (PMC8982894; doi:10.1371/journal.pbio.3001593)
Supplement: S1 Table — p-Values from chi-squared test for PeCoWaCo detection and from Student t test for period comparisons. Red when above 0.05, green when below 0.01, and black in between. See S1 Data for individual quantitative observations. PeCoWaCo, periodic cortical waves of contraction. (DOCX) [file pbio.3001593.s007.docx]

| PeCoWaCo detection (%) | | | | | | | | | | |
| --- | --- | --- | --- | --- | --- | --- | --- | --- | --- | --- |
|  | N total | N osc | **% Osc** | SEM |  |  | Zygote | 2-cell | 4-cell | 8-cell |
| Zygote | 27 | 13 | **48** | 23 | p values | Zygote |  |  |  |  |
| 2-cell | 52 | 18 | **35** | 14 |  | 2-cell | *0.24* |  |  |  |
| 4-cell | 39 | 31 | **79** | 28 |  | 4-cell | *8x10^-3^* | *2x10^-5^* |  |  |
| 8-cell | 34 | 21 | **62** | 24 |  | 8-cell | *0.29* | *0.01* | *0.095* |  |
| PeCoWaCo Period (s) | | | | | | | | | | |
|  | N | mean | **median** | SEM |  |  | Zygote | 2-cell | 4-cell | 8-cell |
| Zygote | 13 | 164 | **147** | 8 | p values | Zygote |  |  |  |  |
| 2-cell | 18 | 126 | **118** | 10 |  | 2-cell | *1x10^-3^* |  |  |  |
| 4-cell | 31 | 84 | **84** | 3 |  | 4-cell | *1x10^-15^* | *8x10^-6^* |  |  |
| 8-cell | 21 | 74 | **74** | 3 |  | 8-cell | *1x10^-15^* | *8x10^-6^* | *0.02* |  |

**S1 Table related to Fig 1**

p values from Chi^2^ test for PeCoWaCo detection and from Student t test for period comparisons. Red when above 0.05, green when below 0.01, black in between. See S1 Data for individual quantitative observations.
