# Supplementary material for: Cortical softening elicits zygotic contractility during mouse preimplantation development
Source: PLoS Biol. 2022 Mar 24;20(3):e3001593. doi: 10.1371/journal.pbio.3001593 (PMC8982894; doi:10.1371/journal.pbio.3001593)
Supplement: S2 Table — p-Values from chi-squared test for PeCoWaCo detection and from Student t test for period comparisons. Red when above 0.05, green when below 0.01, and black in between. See S1 Data for individual quantitative observations. PeCoWaCo, periodic cortical waves of contraction. (DOCX) [file pbio.3001593.s008.docx]

| PeCoWaCo detection (%) | | | | | | | | | | | | |
| --- | --- | --- | --- | --- | --- | --- | --- | --- | --- | --- | --- | --- |
|  | N total | N osc | **% Osc** | SEM |  |  | early-2-cell | late-2-cell | early-4-cell | late-4-cell | early-8-cell | late-8-cell |
| early-2-cell | 33 | 10 | **30** | 14 | p values | early-2-cell |  |  |  |  |  |  |
| late-2-cell | 44 | 9 | **24** | 10 |  | late-2-cell | 0.32 |  |  |  |  |  |
| early-4-cell | 38 | 25 | **66** | 23 |  | early-4-cell |  |  |  |  |  |  |
| late-4-cell | 29 | 19 | **66** | 24 |  | late-4-cell |  |  | *0.27* |  |  |  |
| early-8-cell | 17 | 8 | **47** | 25 |  | early-8-cell |  |  |  |  |  |  |
| late-8-cell | 26 | 15 | **58** | 25 |  | late-8-cell |  |  |  |  | 0.49 |  |
| PeCoWaCo Period (s) | | | | | | | | | | | | |
|  | N | mean | **median** | SEM |  |  | early-2-cell | late-2-cell | early-4-cell | late-4-cell | early-8-cell | late-8-cell |
| early-2-cell | 11 | 136 | **147** | 12 | p values | early-2-cell |  |  |  |  |  |  |
| late-2-cell | 9 | 129 | **118** | 20 |  | late-2-cell | *0.76* |  |  |  |  |  |
| early-4-cell | 27 | 92 | **84** | 5 |  | early-4-cell |  | *0.01* |  |  |  |  |
| late-4-cell | 17 | 77 | **74** | 2 |  | late-4-cell |  |  | *0.03* |  |  |  |
| early-8-cell | 8 | 73 | **74** | 6 |  | early-8-cell |  |  |  | *0.36* |  |  |
| late-8-cell | 15 | 75 | **74** | 4 |  | late-8-cell |  |  |  |  | *0.85* |  |

**S2 Table related to S1 Fig**

p values from Chi^2^ test for PeCoWaCo detection and from Student t test for period comparisons. Red when above 0.05, green when below 0.01, black in between. See S1 Data for individual quantitative observations.
