# Supplementary material for: Cortical softening elicits zygotic contractility during mouse preimplantation development
Source: PLoS Biol. 2022 Mar 24;20(3):e3001593. doi: 10.1371/journal.pbio.3001593 (PMC8982894; doi:10.1371/journal.pbio.3001593)
Supplement: S3 Table — p-Values from chi-squared test for PeCoWaCo detection and from Student t test for period comparisons. Red when above 0.05, green when below 0.01, and black in between. See S1 Data for individual quantitative observations. PeCoWaCo, periodic cortical waves of contraction. (DOCX) [file pbio.3001593.s009.docx]

| PeCoWaCo detection (%) | | | | | |
| --- | --- | --- | --- | --- | --- |
|  | N total | N osc | **% Osc** | SEM | p |
| DMSO | 20 | 18 | **90** | 6.67 |  |
| Vx-680 | 12 | 9 | **75** | 9.69 | *0.258* |
| PeCoWaCo Period (s) | | | | | |
|  | N | mean | **median** | SEM | p |
| DMSO | 18 | 92.05 | **3.06** | 91.04 |  |
| Vx-680 | 9 | 96.56 | **9.14** | 84.03 | *0.54* |
|  |  |  |  |  |  |
| PeCoWaCo detection (%) | | | | | |
|  | N total | N osc | **% Osc** | SEM | p |
| Mech Control | 14 | 6 | **42.86** | 23.2 |  |
| Fragmented Cell | 14 | 7 | **50** | 23.33 | *0.7* |
| Enucleated Fragment | 14 | 7 | **50** | 14.43 | *0.7* |
| PeCoWaCo Period (s) | | | | | |
|  | N | mean | **median** | SEM | p |
| Mech Control | 6 | 127.42 | **122.55** | 27.57 |  |
| Fragmented Cell | 7 | 117.86 | **117.65** | 20.47 | *0.76* |
| Enucleated Fragment | 7 | 149.86 | **147.06** | 18.87 | *0.47* |

**S3 Table related to Fig 2**

p values from Chi^2^ test for PeCoWaCo detection and from Student t test for period comparisons. Red when above 0.05, green when below 0.01, black in between. See S1 Data for individual quantitative observations.
