# Supplementary material for: Cortical softening elicits zygotic contractility during mouse preimplantation development
Source: PLoS Biol. 2022 Mar 24;20(3):e3001593. doi: 10.1371/journal.pbio.3001593 (PMC8982894; doi:10.1371/journal.pbio.3001593)
Supplement: S4 Table — p-Values from Student t test. Red when above 0.05, green when below 0.01, and black in between. See S1 Data for individual quantitative observations. (DOCX) [file pbio.3001593.s010.docx]

| Surface tension (pN/µm) | | | | | |
| --- | --- | --- | --- | --- | --- |
|  | N | mean | **median** | SEM | p |
| DMSO | 12 | 364 | **385** | 32 |  |
| Vx-680 | 13 | 290 | **241** | 52 | *0.14* |
|  |  |  |  |  |  |
| Surface tension (pN/µm) | | | | | |
|  | N | mean | **median** | SEM | p |
| Mech Control | 14 | 1643 | **1244** | 299 |  |
| Fragmented Cell | 14 | 1864 | **1836** | 236 | *0.89* |

**S4 Table related to S2 Fig**

p values from Student t test. Red when above 0.05, green when below 0.01, black in between. See S1 Data for individual quantitative observations.
