# Supplementary material for: Cortical softening elicits zygotic contractility during mouse preimplantation development
Source: PLoS Biol. 2022 Mar 24;20(3):e3001593. doi: 10.1371/journal.pbio.3001593 (PMC8982894; doi:10.1371/journal.pbio.3001593)
Supplement: S5 Table — p-Values from Student t test. Red when above 0.05, green when below 0.01, and black in between. See S1 Data for individual quantitative observations. (DOCX) [file pbio.3001593.s011.docx]

| PeCoWaCo Period (s) | | | | | | | | | |
| --- | --- | --- | --- | --- | --- | --- | --- | --- | --- |
|  | N | mean | **median** | SEM |  |  | 2 x 1/16th | 4 x 1/16th | 8 x 1/16th |
| 2 x 1/16th | 18 | 97.7 | **85.14** | 7.72 | p values | 2 x 1/16th |  |  |  |
| 4 x 1/16th | 22 | 95.58 | **84.88** | 4.52 |  | 4 x 1/16th | *0.8* |  |  |
| 8 x 1/16th | 11 | 104.18 | **100** | 5.44 |  | 8 x 1/16th | *0.54* | *0.24* |  |
| PeCoWaCo velociity | | | | | | | | | |
|  | N | mean | **median** | SEM |  |  | 2 x 1/16th | 4 x 1/16th | 8 x 1/16th |
| 2 x 1/16th | 18 | 0.45 | **0.42** | 0.02 | p values | 2 x 1/16th |  |  |  |
| 4 x 1/16th | 23 | 0.44 | **0.43** | 0.01 |  | 4 x 1/16th | *0.98* |  |  |
| 8 x 1/16th | 11 | 0.46 | **0.46** | 0.02 |  | 8 x 1/16th | *0.64* | *0.51* |  |
|  |  |  |  |  |  |  |  |  |  |
| PeCoWaCo Period (s) | | | | | | | | | |
|  | N | mean | **median** | SEM |  |  | Mech Control | Fragmented Cell | Enucleated Fragment |
| Mech Control | 6 | 85.06 | **84.77** | 3.81 | p values | Mech Control |  |  |  |
| Fragmented Cell | 8 | 91.95 | **100** | 7.89 |  | Fragmented Cell | *0.55* |  |  |
| Enucleated Fragment | 4 | 88.41 | **85.14** | 4.57 |  | Enucleated Fragment | *0.46* | *0.75* |  |
| PeCoWaCo velocity | | | | | | | | | |
|  | N | mean | **median** | SEM |  |  | Mech Control | Fragmented Cell | Enucleated Fragment |
| Mech Control | 6 | 0.48 | **0.48** | 0.04 | p values | Mech Control |  |  |  |
| Fragmented Cell | 8 | 0.42 | **0.43** | 0.02 |  | Fragmented Cell | *0.69* |  |  |
| Enucleated Fragment | 4 | 0.45 | **0.46** | 0.06 |  | Enucleated Fragment | *0.13* | *0.47* |  |

**S5 Table related to Fig 3**

p values from Student t test. Red when above 0.05, green when below 0.01, black in between. See S1 Data for individual quantitative observations.
