# Supplementary material for: Cortical softening elicits zygotic contractility during mouse preimplantation development
Source: PLoS Biol. 2022 Mar 24;20(3):e3001593. doi: 10.1371/journal.pbio.3001593 (PMC8982894; doi:10.1371/journal.pbio.3001593)
Supplement: S6 Table — p-Values from Student t test. Red when above 0.05, green when below 0.01, and black in between. See S1 Data for individual quantitative observations. (DOCX) [file pbio.3001593.s012.docx]

| Surface tension (pN/µm) | | | | | | | | | |
| --- | --- | --- | --- | --- | --- | --- | --- | --- | --- |
|  | N | mean | **median** | SEM |  |  | 2 x 1/16th | 4 x 1/16th | 8 x 1/16th |
| 2 x 1/16th | 14 | 492.1 | **579.03** | 51.11 | p values | 2 x 1/16th |  |  |  |
| 4 x 1/16th | 20 | 378.54 | **364.73** | 46.18 |  | 4 x 1/16th | *0.1* |  |  |
| 8 x 1/16th | 18 | 352.83 | **287.22** | 55.11 |  | 8 x 1/16th | *0.07* | *0.71* |  |

**S6 Table related to S3 Fig**

p values from Student t test. Red when above 0.05, green when below 0.01, black in between. See S1 Data for individual quantitative observations.
