# Supplementary material for: Cortical softening elicits zygotic contractility during mouse preimplantation development
Source: PLoS Biol. 2022 Mar 24;20(3):e3001593. doi: 10.1371/journal.pbio.3001593 (PMC8982894; doi:10.1371/journal.pbio.3001593)
Supplement: S8 Table — p-Values from chi-squared test for PeCoWaCo detection and from Student t test for period and surface tension comparisons. Red when above 0.05, green when below 0.01, and black in between. See S1 Data for individual quantitative observations. PeCoWaCo, periodic cortical waves of contraction. (DOCX) [file pbio.3001593.s014.docx]

| Cortex Thickness (µm) | | | | | | | | | | |
| --- | --- | --- | --- | --- | --- | --- | --- | --- | --- | --- |
|  | N | mean | **median** | SEM |  |  | Zygote | 2-cell | 4-cell | 8-cell |
| Zygote | 15 | 0.49 | **0.48** | 0.02 | p values | Zygote |  |  |  |  |
| 2-cell | 19 | 0.57 | **0.58** | 0.02 |  | 2-cell | *0.013* |  |  |  |
| 4-cell | 13 | 0.49 | **0.51** | 0.03 |  | 4-cell | *0.82* | *0.02* |  |  |
| 8-cell | 11 | 0.5 | **0.47** | 0.02 |  | 8-cell | *0.77* | *0.04* | *0.65* |  |

**S8 Table related to S5 Fig**

p values from Chi^2^ test for PeCoWaCo detection and from Student t test for period and surface tension comparisons. Red when above 0.05, green when below 0.01, black in between. See S1 Data for individual quantitative observations.
