# Supplementary material for: Cortical softening elicits zygotic contractility during mouse preimplantation development
Source: PLoS Biol. 2022 Mar 24;20(3):e3001593. doi: 10.1371/journal.pbio.3001593 (PMC8982894; doi:10.1371/journal.pbio.3001593)
Supplement: Attachment — Submitted filename: 118346_1_rebuttal_2180637_qzqbfm.docx [file pbio.3001593.s027.docx]

**Manuscript number:** RC-2021-00931

**Corresponding author(s):** Jean-Léon Maître

## 1. General Statements [optional]

We thank both reviewers for carefully evaluating our study and for their constructive comments and clear suggestions. We are also grateful for their supportive appreciation of our study and for noting that it could have a wide impact in several fields of biology.

## 2. Description of the planned revisions

#1 Both reviewers pointed at the different analytical techniques, namely PIV and curvature tracking, used to characterize periodic contractions in Fig3.

This raised questions regarding the rationale for not using the same analysis throughout our study. We also noted a confusion resulting from our mistake of not providing images of periodic contractions without the results of the PIV analysis overlaid on them. We want to clarify that periodic cortical waves of contraction (PeCoWaCo) occur at the cortex of cells without any obvious cytoplasmic streaming associated with them, unlike, for example, the pulsed contractions observed in *c. elegans* zygotes (Munro et al, 2004 or Mayer et al 2010). Therefore, PIV analysis captures PeCoWaCo movements from the deformation of cell surface and associated cell and embryo jiggling but not from cytoplasmic movement. **We will provide movies of PeCoWaCo without the arrows resulting from PIV analysis to clarify the confusion over the presence of cytoplasmic streaming**. Curvature tracking accurately captures the surface deformation of cells during PeCoWaCo and is a more accurate technique than PIV to characterize the amplitude of deformations and the travelling velocity of the wave. However, it is not as sensitive as PIV at detecting PeCoWaCo when this phenomenon is present in a subtle manner. Therefore, we have used PIV when investigating the presence or absence of PeCoWaCo in different conditions (Fg1-2 and 4) and curvature tracking when studying the properties of PeCoWaCo (Fig3). Nevertheless, to clarify any doubt about the use of either analytical techniques, **we will provide measurement using both approaches, as requested by reviewers**.

#2 Both reviewers raised concerns over the absence of surface tension measurements as a control of the experiments in which we manipulate cell size. We have carried out these measurements and can confirm that manipulating cell size does not change surface tension or as, we had described in our first submission, PeCoWaCo properties. **We will provide surface tension measurements of size-manipulated embryos**. Similarly, reviewers wanted to see surface tension measurements of embryos treated with Latrunculin A. We have carried out those measurements and can also confirm that, as expected from previous studies including in mouse preimplantation embryos (Maître et al, 2015), inhibition of actin polymerisation decreases surface tension. **We will provide surface tension measurements of Latrunculin A treated embryos**.

#3 Both reviewers felt that the molecular changes associated with cortical behaviors during cleavage stages ought to be better characterized. We have further characterized the structure and molecular composition of the cortex during cleavage stages. **We will provide cortex thickness measurements, as requested by Reviewer#1**. We have also carried out **immunostaining of cortical components** (actin regulators and myosin motors), **which we will provide** to better characterize the molecular composition of the cortex during cleavage stages. In addition, **we will provide analyses cortical components expression from available proteomics data on preimplantation mouse embryos,** which we used to identify Fmnl3 as a potential candidate for controlling cortical softening during cleavage stages. Fmnl3 is a formin whose levels significantly drop during cleavage stages. As a formin, it could be involved in cortical actin remodeling. We have confirmed the cortical localization of Fmnl3 during cleavage stages and are currently testing how maintaining its expression affects cleavage stages. **We will provide Fmnl3 localization and overexpression data**. In addition to Fmnl3 overexpression, to increase actin stability, **we will also provide analyses of Jasplakinolide treated embryos**. Finally, reviewers requested analysis of upstream contractility regulators inhibition. **We will provide analysis of Rho inhibition** and discuss the use of other inhibitors of contractility regulators that we have tested (myosin inhibitors).

#4 Finally, we will address the other important minor points raised by Reviewer#1 concerning the relationship of periodic contractions with the cell cycle by **providing movies of cells in division**. **We will provide complete statistical analyses of the different conditions mentioned by Reviewer#1**. Moreover, **we will provide our raw data on a dedicated server**, as we had done with previous studies from the lab (Dumortier et al 2019 and Schliffka, Tortorelli et al 2021).

## 3. Description of the revisions that have already been incorporated in the transferred manuscript

The current manuscript is not different from the original submission.

## 4. Description of analyses that authors prefer not to carry out

#1 Among the experiments suggested by Reviewer#1 to better characterize the cortex of cleavage stage embryos is FRAP to analyze actin dynamics. We have been experiencing technical difficulties to obtain embryos expressing actin-gfp with sufficient signal for FRAP analysis without compromising viability. We do not think we can make this work within a reasonable time. We hope that the current (surface tension, PeCoWaCo) and additional (thickness, molecular composition) characterizations of the cortex will suffice to better understand the cortical changes occurring during cleavage stages.

#2 In addition to reducing tension, Reviewer#1 suggested to increase it using Concanavalin A, agarose or collagen embedding. We do not think these treatments would lead to a tension increase and, from our experience, it is difficult to grow healthily developing mouse embryos in those conditions. We hope the overexpression of Fmnl3 and Jasplakinolide treatment will serve as good alternative to Reviewer#1’s suggestions.
