# Supplementary material for: Cortical softening elicits zygotic contractility during mouse preimplantation development
Source: PLoS Biol. 2022 Mar 24;20(3):e3001593. doi: 10.1371/journal.pbio.3001593 (PMC8982894; doi:10.1371/journal.pbio.3001593)
Supplement: Attachment — Submitted filename: 220106ResponseReferees.docx [file pbio.3001593.s029.docx]

We want to thank both reviewers for their constructive criticism and insightful suggestions.

Before addressing each of their points individually, we would like to notify important changes in the methods of our study that were not solicited during the initial reviewing process.

1-We have discovered a bug in the acquisition software of our microscope. This resulted in the two initial frames of our time lapses to be taken every 3 s instead of the requested 5 s steps. The following 118 steps would be correctly taken every 5 s. Since the Fourier transform that we have used to characterize PeCoWaCo is sensitive to the framerate and duration of sampling, we have re-analyzed all of the data, excluding the 2 first time points that were taken with an incorrect delay. All of the PeCoWaCo graphs have been updated, even if at first glance they may appear identical to the previous ones.

2-We have taken advantage of this situation to improve our analysis and make the classification of our samples more objective and reproducible. To objectively determine the threshold value for classifying whether a given sample oscillates or not, we have adopted a statistical method based on logistic regression (see methods). This has changed the threshold value for classifying cells and embryos as oscillating from 1.85 to 1.777 (Fig S1).

The modifications of the image analysis and classification pipeline have slightly modified some of the graphs and statistics. The most noticeable changes are the amplitude values after Fourier analysis and the oscillation period for the zygote stage. However, **none of the conclusions of our study have been modified due to this change**.

Reviewer #1 (Evidence, reproducibility and clarity (Required)):

Periodic waves of contraction (PeCoWaCo) are stereotypical phenomena conserved in many oocytes and embryos that can lead to large-scale shape changes. However, their function is often unclear, and the parameters controlling their characteristics (de novo onset, amplitude, velocity...) still elusive in many model systems. In this manuscript, Ozguc and collaborators study the initiation of zygotic contractility during preimplantation development in the mouse. They show that PeCoWaCo become detectable in early embryos after the 2-cell stage, and progressively increase in frequency with each successive cleavage. Using elegant and challenging macro-manipulations of mouse embryos, they show that PeCoWaCo are not initiated by a reduction in cell size inherent in cell cleavage, nor their oscillation properties. Instead, PeCoWaCo are initiated and amplified by a gradual decrease in surface tension from the zygote to the 8-cell stage. The authors suggest that cortical softening awakens zygotic contractility.

The question addressed by the authors is an important one, in several fields (embryogenesis, cell biology, biophysics) and could therefore have wide impact. However, although the data presented are convincing, they fall short, controls are lacking to fully support the conclusions, and easy experiments could complement the existing limited results and take the paper to a higher level, making it a more complete study.

We thank the reviewer for carefully reviewing our study and for their constructive comments and suggestions.

**Major comments:**

-Since the authors talk about "cortical maturation" during early embryogenesis and briefly about actin polymerization, they should describe the cortex itself during early embryogenesis (thickness, composition in terms of actin nucleators, actin dynamics by FRAP...). This could also help to understand why embryos decrease their surface tension during early development.

We agree with the reviewer that better characterizing the cortex during its maturation is important. Following the reviewer’s suggestion, we have used super-resolution microscopy and measured the thickness of the cortex. **We now provide cortex thickness measurements in Fig S5.** This reveals a transient cortical thickening at the 2-cell stage, which may be hinting further at cortical maturation during cleavage stages.

Also, we tested the localization of several actin binding proteins by immunostaining to see how the cortex may change composition during cleavage changes. **We now provide immunostaining of actin binding proteins during cleavage stages in Fig S5.**

In addition, **we now provide analyses of the expression of actin binding proteins such as arps and formins from available single cell mRNA sequencing and proteomics data on preimplantation mouse embryos (Fig S5)**.

Among the experiments suggested by the Reviewer to better characterize the cortex of cleavage stage embryos is FRAP to analyze actin dynamics. We have been experiencing technical difficulties to obtain cleavage stage embryos expressing actin-GFP with sufficient signal for FRAP analysis without compromising viability. We do not think we can make this work within a reasonable timeframe. We hope that the initial (surface tension, PeCoWaCo) and additional (thickness, molecular composition, expression data) characterizations of the cortex will suffice to better describe the cortical changes occurring during cleavage stages.

-The authors use only one mean to reduce the tension of the cortex, using low doses of Latrunculin A. First, the authors should actually show that surface tension is reduced after this treatment by measuring it by micropipette aspiration, as they do in Figure 4 for control embryos, and show actin organization after such treatment (by immunofluorescence or live imaging). Second, could they use other means to reduce the tension of the cortex (for example play with actin polymerization by addressing actin nucleators/regulators at the cortex, or play with Myosin-II activity...)? Third, could they do the reverse, increase the tension of the cortex after the 2-cell stage to test if this inhibits/decreases PeCoWaCo (using Concanavallin A as in Kunda 2008, or embedding the embryos in loose collagen, or attracting Myosin-II to the cortex...)? Finally, what is the long-term effect of disrupting the appearance/characteristics of PeCoWaCo on subsequent embryo development? This question could be addressed by tuning the intensity of PeCoWaCo using the experiments suggested above in the zygote or at the 2-cell stage, and monitoring embryo development up to the blastocyst stage to assess the outcome on embryo development. 

We have measured the surface tension of Latrunculin A-treated embryos and confirm that they have reduced surface tension. **We now provide surface tension measurements of Latrunculin A treated embryos in Fig 4**.

Other attempts to reduce tension by inhibiting cell contractility resulted in the absence of PeCoWaCo, even when trying to carefully titrate the concentrations of drugs such as Blebbistatin or ML7. In addition, we had previously analyzed PeCoWaCo of mutants of Myh9 and/or Myh10, which encode distinct non-muscle myosin II heavy chain paralogs. These mutants rarely showed detectable PeCoWaCo (Schliffka, Tortorelli, 2021). We suspect that myosin activity is essential for the generation of PeCoWaCo, which makes myosin loss of function an unsuitable approach to study PeCoWaCo properties in the mouse embryo. On the other hand, disrupting the structure of the actin meshwork, for example as a result of Latrunculin A scavenging G-actin leading to global shortening of actin filaments, still permits PeCoWaCo to appear with modified properties.

As far as tension increase is concerned, **we have injected embryos with GFP-Fmnl3 mRNA, encoding a formin whose expression decays during cleavage stages, and find that this is sufficient to raise surface tension and delay the appearance of PeCoWaCo (Fig 5).** The effect of Fmnl3 overexpression is transient: embryos show PeCoWaCo at the end of the 4-cell stage and develop to blastocyst normally (Fig S6, Movie 11). This could be due to the loss of GFP-Fmnl3, which would mirror the one of the endogenous Fmnl3 mRNA and protein (Fig S5).

In addition, the Reviewer suggested to increase surface tenson using Concanavalin A, agarose or collagen embedding. We do not think these treatments would lead to a tension increase and, based on our experience, it is difficult to grow healthily developing mouse embryos in those conditions. We hope the overexpression of Fmnl3 will serve as good alternative to these suggestions.

-In most figures, with the exception of Figure 3, the authors detect and analyze PeCoWaCo using PIV, a technique that tracks movements in the cytoplasm. However, cytoplasmic movements are not always due to the contractility of the cortex and PeCoWaCo. They can be generated by cortex-independent cytoplasmic activity. Thus, it would have been preferable to follow the cell contour as in Figure 3 for all figures, for all stages (on dissociated embryos for 4 and 8-cell stages if too challenging due to overlapping of blastomeres in 2D). Another way to validate PIV as a means of detecting PeCoWaCo without having to repeat all the experiments could be to correlate PIV with oocyte contour in control embryos at all stages.

We realize that we have not provided movies of embryos without the PIV arrows overlaid on them. This has caused some confusion since there is in fact very little, if any, cytoplasmic movement. We apologize for this misunderstanding.

PIV correlates clusters of pixels between successive time points and tracks any kind of movements. When the surface of the cells contracts periodically, it moves the cells which displaces clusters of pixels periodically. However, there is no cytoplasmic streaming such as the ones observed, for example, in mouse oocytes or c elegans zygotes. **We now provide movies of PeCoWaCo without the arrows resulting from PIV analysis to make it easier to appreciate the absence of cytoplasmic streaming (Movie 1)**.

The reason we have used PIV is that it is more sensitive than contour tracking at detecting weak oscillations. Therefore, this is the best method to identify the proportion embryos showing oscillations and we have limited our contour tracking to cases when we wanted to analyze the wave velocity in addition to its period (Fig 3). Nevertheless, we agree that the comparison of the methods is relevant to the understanding of the study and **we now provide a side by side comparison of both methods (Fig S4, Movie 5)**. We have applied the contour analysis to some of the embryos we had previously only analyzed with PIV. As explained, contour analysis is less sensitive than PIV at detecting PeCoWaCo but, when detected with both approaches, the oscillations have the same period indicating that PIV indeed detects movements that are due to the cortical deformations.

-In the treatments perturbing the cell size to test whether it is sufficient to initiate PeCoWaCo (Figure 2) or modify their periodicity (Figure 3), how is the surface tension between all conditions? Indeed, the authors nicely show that the cell size does not matter for PeCoWaCo, but that the cell surface does matter, so if PeCoWaCo are similar between cells of different sizes, it means that their surface tension should be in the same range as well. The authors should therefore measure it by micropipette aspiration.

We agree that this is an important control. **We now provide surface tension measurements of size-manipulated embryos (Fig S2-3)** and we can confirm that, like the oscillation period, surface tension is not affected by cell size manipulations.

- re the characteristics of the PeCoWaCo the same in interphase and mitosis? In Bischof 2017, surface contraction waves in starfish oocytes are regulated by a cdk1-cyclinB gradient in meiosis. Could such a mechanism play a role in what the authors observe in early mouse embryos? For example, is there a correlation between cell cycle length (and especially M-phase) and PeCoWaCo strength? If so (shorter cell cycles/M-phases in zygotes compared to 8-cell stage embryos), one could imagine that mouse embryos are progressively exposed to cdk1-cyclinB for longer periods of time during their development, which would contribute to the appearance and increase in PeCoWaCo.

This may be an important difference between PeCoWaCo and the contraction waves observed in frog and starfish oocytes. In preimplantation mouse embryos, PeCoWaCo are specific to the interphase. **To illustrate this, we now provide movies of cells during mitosis (Movie 2).** We do not see or detect PeCoWaCo during mitosis. Therefore our analysis has focused on the interphase. We have clarified this in the Methods section.

Regarding the duration of Cdk1 exposure across cleavage stages, the zygote and 2-cell stage last longer than the subsequent cleavage stages. The zygote stage has a longer G1, during which pronuclei meet, and G2, during which the minor ZGA occurs. The 2-cell stage has a longer G2, during which the major ZGA occurs. Based on the literature, cells then spend most of their 4- and 8-cell stages in S phase with a rather short G2/M (Artus, Cohen Tannoudji 2008). Therefore, it seems unlikely that PeCoWaCo would be related to longer exposure to Cdk1-CyclinB during G2/M with succeeding cleavage stages.

**Minor comments:**

-Why does the proportion of embryos with oscillations decrease at the 8-cell stage compared to the 4-cell stage (Figure 1D)? It should not, since the surface tension is lower in 8-cell stage compared to the 4-cell stage (Figure 4B).

We believe this is in part due to PIV analysis. We have used the same interrogation window size for all conditions. Therefore, with cells becoming progressively smaller with each cleavage, we sample each cell proportionally less and less with each cleavage division.

An additional explanation could be the start of compaction at the 8-cell stage, which reduces contrast at cell boundaries. This makes movement less detectable to PIV. For this reason, we have focused our analysis on pre-compaction 8-cell stage embryos, as stated in the Methods section.

-Figure 2G shows that PeCoWaCo are not detected in either control or fragmented cells at the 2-cell stage but with a volume corresponding to a 4-cell stage, suggesting that 4-cell stage blastomere size is not sufficient to trigger PeCoWaCo. However, in Figure 1D, PeCoWaCo are detected in half of the control embryos at the 2-cell stage. Why is there a difference in the % of control embryos at the 2-cell stage with oscillations (0% versus 50%) between these 2 experiments? Could this be due to the CCD treatment allowing volume reduction (but also maybe reduction in surface tension) in Figure 2G?

In Fig 1D, we had sampled each embryos 2-5 times throughout their 2-cell stage, noting them as showing PeCoWaCo if any of the individual sampling turned positive. In the fragmentation experiment initially shown in Fig 2G, we had sampled the cells only once, which could explain the lower detection rate in the control. **We now have sampled the fragmented cells 3 times and detect oscillations in ~50% of them, which is in better agreement with Fig 1D.**

We do not think that CCD treatment, applied to control cells as well as to fragmented ones 1 h before imaging, influence their ability to generate PeCoWaCo. Our reasoning is that the same treatment applied to fragment 16-cell stage blastomeres did not prevent cells or fragments from robustly generating PeCoWaCo within 1 h, as shown in Fig 3.

-Since the authors fuse and dissociate embryos at the 16-cell stage, they should show the period and velocity of PeCoWaCo in unmanipulated 16-cell stage embryos prior to any treatment.

**We now provide the properties of isolated 16-cell stage blastomeres in Fig 3C-D.**

-In Figure 3, the percentage of embryos with detected oscillations should be added to the figure, as for Figure 1D or 2G.

For Fig 3, we did not aim at understanding how cell size influence the presence of PeCoWaCo but rather how cell size may influence the oscillatory properties of the travelling waves. Therefore, we have only analyzed cells that do show oscillation and **we cannot give the percentage of oscillating cells in this case**. We have clarified this in the dedicated methods section.

-In Figure 3C-F, the period of the PeCoWaCo is about 100 s, higher than that of the 8-cell stage in Fig1E, which is about 75 s. Is this difference statistically significant? And if so, why would it be higher in Figure 3? 

As far as statistical tests are concerned, we do not think that comparing samples that were acquired in entirely different settings would be rigorously correct. That being said, the higher period of isolated 16-cell stage blastomeres seems real. We do not know whether this difference could be due to the dissociation process.

-Since PeCoWaCo become detectable after the 2nd cleavage, and thus after the ZGA, it could depend on a development program, allowing the surface tension to be reduced. The authors could have discussed this point further in the discussion

**We have extended our discussion on the ZGA.**

Finally, the figures are difficult to read and the legends lack precision: 
-Some x-axis should be more informative with more than 3 values (Figures 1D, 2C, 2G, 4D, S1A).

- he value of the averages, or the exact percentages, should appear in full somewhere in the text, figures or legends (Figures 1D, 1E, 2C, 2D, 2G, 3C, 3D, 3F, 3G, 4B, 4D, 4E, S1A, S1B, S2).

-The exact p-value should be written in full somewhere in the text, figures or legends (concerns all figures). 

We think adding more values on the y axis makes the graph difficult to read. Since we also indicate the sample sizes, we think the correct level of information is given for readers to evaluate the proportion of oscillating cells and embryos. **We now provide tables containing mean, median, SEM, n and p values that are relevant to the study in a dedicated supplementary table (Table 1-9)**. Moreover, **we will provide our raw data to download freely from a dedicated server**, as we had done with previous studies from the lab (Dumortier et al 2019 and Schliffka, Tortorelli et al 2021).

-Sometimes the titles of the axes do not correspond to what the graph shows. For example, in graphs 1D, 2C, 2G, 4D, S1A, the x-axis shows all the embryos, not just the ones with oscillations.

The axes of the graphs now in Fig 1D, 2C, 2G, 4F and S1G do indicate the percentage of embryos with detected oscillations.

-some figure legends lack information: What are the white arrows in Figure 3A? What are we looking at in Figure 3A, embryos in transmitted light or expressing a probe to follow the embryo contour? T and V in Figure 3A represent period and velocity, but it is not stated in the legend. The curvature bar in Figure 3A is strange as it is mostly white. The scale bar values are missing for Figures 2B, 2F, 4A and 4C.

We apologize for the lack of information and thank the reviewer for bringing this to our attention. **We have added the relevant descriptions**: the white arrows are pointing at the flat part of the cell surface corresponding to the passing of the contractile wave, the image shows a blastomere expressing a fluorescent membrane marker, T and v are indeed the period and velocity of the waves. **We have modified the scales** for curvature and sizes.

-In Figure 3, the y-axes are not all consistent, sometimes the radius of the embryos is shown, sometimes fractions of 16-cell stage cells (which is not easy to read).

We apologize for the confusion. The “1/16^th^” nomenclature is commonly used when describing a blastomere coming from dissociated 16-cell stage mouse embryo. We fused varying numbers of these isolated blastomeres resulting in distinct experimental conditions (“8 x”, “4 x” and “2 x 1/16^th^”). Fusing several of these blastomeres results in larger cells with slightly varying radii in each condition. Similarly for fragmentation, fragments do not always have the same size but reflect the same experimental conditions. The graphs shown in Fig 3F-G, 3I-J reflect these distinct experimental conditions. On the other hand, Fig 3K-L are an attempt to group these experimental conditions together using cell radii to get a general impression on the (lack of) relationship between oscillation properties and cell size. We think both representations serve different purposes, which make up for the decrease in readability.

Reviewer #1 (Significance (Required)):

The question addressed by the authors is an important one, in several fields (embryogenesis, cell biology, biophysics) and could therefore have wide impact. However, although the data presented are convincing, they fall short, controls are lacking to fully support the conclusions, and easy experiments could complement the existing limited results and take the paper to a higher level, making it a more complete study.

We thank once more the reviewer for being supportive and constructive. We hope our responses and additions to the manuscript will clear their concerns.

Reviewer #2 (Evidence, reproducibility and clarity (Required)):

The manuscript by Özgüc and coworkers analyses the cortical contraction waves that are observed in early mouse embryos around the 8- to 16-cell stage. First, they provide a very careful, precise quantitative characterization of these contraction waves using PIV (particle image velocitometry) and by measuring the local cortex curvature. They find that contraction waves 'awaken' as development progresses, i.e. the oscillation frequency (and amplitude) of the waves is increasing during the cleavage divisions.

Then, they use a number of very elegant micromanipulations to dissect whether the decreasing cell size is the cause for the increased cortical activity and increased frequency of contraction waves. Somewhat surprisingly, but very clearly they demonstrate that cortical contractions are independent of cell size. These results are consistent across a wide range of manipulated cell (fragment) sizes, and various types of treatments.

Next, they measure the surface tension of cells using micropipette suction. These experiments reveal a clear correlation between surface tension and the period of contraction waves. To prove the causal relation between these processes, they artificially decrease the surface tension in 2-cell embryos by partially depolymerizing the cortex using Latrunculin A. This led to a strengthening of the contractions.

Together, the authors conclude that properties of cortical contraction waves are independent of cell size. Instead, they propose that the reason for the gradually increasing frequency of cortical contraction waves is the gradual softening of the cell cortex accompanying cleavage divisions.

We thank the reviewer for their appreciation of our study.

**Major comments:**

1.The authors start introducing contraction waves by quantifying cytoplasmic flows using PIV. I would find it important to first show that deformations of the cortex are indeed causing the observed cytoplasmic flow patterns, which should be straightforward to analyze by using the tools shown on Fig. 3A. As there are other known, cortex independent mechanisms to generate cytoplasmic flows, these experiments would be important to exclude such alternative mechanisms.

We want to clarify a confusion caused by our mistake not to provide movies of PeCoWaCo without PIV arrows overlaid on them. There is in fact very little, if any, cytoplasmic movement. We apologize for this misunderstanding.

PIV correlates clusters of pixels between successive time points and tracks any kind of movements. When the surface of the cells contracts periodically, it moves the cells which displaces clusters of pixels periodically. However, there is no cytoplasmic streaming such as the ones observed in mouse oocytes or c elegans zygotes for example. **We now provide movies with and without PIV overlay to make the phenomenon clearly visible to the readers (Movie 1).**

2.I am very impressed by the various manipulations the authors use to demonstrate the independence of cell size and cortical contractions. However, I wonder why these manipulations have not been used in combination with surface tension measurements. Even more critically, I am missing more experiments that would directly demonstrate the causal relationship between cortical tension and contraction period. Unfortunately, the Latrunculin A results are less clear (as compared to other results shown in the paper), and the effect may indeed be more complex. For example, Bement et al (NCB, 2015) suggested a biochemical feedback mechanism based on F-actin, which in this experiment could not be distinguished from 'mechanical' effects of surface tension. Generally, I feel that additional perturbation experiments are needed in order to strengthen the conclusions. This should include upstream pathways known to regulate cortical contractility such as RhoA, for example.

In light of our findings, we agree that surface tension measurements of cells with manipulated sizes are important. **We have added those measurements (Fig 4-5, S2-3).**

As far as further characterization of the causal relationship between tension and contraction period, we have inhibited RhoA as suggested by the reviewer. Adding C3 transferase to cleavage stage embryos or to 16-cell stage embryos showed no effect on PeCoWaCo. It did affect compaction and blastocyst formation, suggesting that the compound was functional.

Based on the literature on pulsed contraction and the PeCoWaCo, we believe it is unlikely that they would not rely on Rho activity. Therefore, we are reluctant to conclude from these experiments. We believe that further experiments would be needed to clarify the role of Rho. However, these experiments would require a significant amount of time and the purpose of this study is not exhaustively dissect the signaling pathway controlling PeCoWaCo. We think the present manuscript already provides enough novel observations and mechanisms to develop an interesting narrative.

Further molecular characterization of the cortex during cleavage stages led us to investigate Fmnl3, a formin, which levels drop during cleavage stages. Artificially maintaining the expression of Fmnl3 by mRNA injection is sufficient to maintain surface tension at higher levels and to delay the appearance of PeCoWaCo (Fig 5).

**Minor comment:**

I would not mind if the authors used PeCoWaCo as an acronym through the present manuscript, but as they state, this is likely to be a very conserved feature of the cell cortex possibly of all animal cells. With this in mind, I doubt that the community would adopt such a rather complicated acronym to describe this process.

By no mean do we intend to push the PeCoWaCo acronym onto the community interested in cortical waves or periodic contractions. As mentioned throughout the present discussion, we actually think that, while PeCoWaCo share some similarities with other contractile waves and periodic surface waves observed in other settings, they are different in several ways. We think periodic cortical waves of contraction is a fitting definition for the present phenomenon, and PeCoWaCo is simply a shorter way of mentioning them.

Reviewer #2 (Significance (Required)):

Waves of cortical contractions are likely to be a widely conserved feature of the cortex of animal cells. The present manuscript makes important contributions to understand this process by (i) developing tools to quantify the process, (ii) show that contraction waves are independent of cell size, (iii) the period of contractions likely depend on the surface tension of the cell. These findings are broadly relevant for the cell biology community as a whole, however, as stated above, the findings needs to be substantially strengthened before publication, in my opinion.

We are grateful to the Reviewer for appreciating the relevance of our study and we hope we can strengthen our conclusion in line with their wishes.
