# Supplementary material for: Cortical softening elicits zygotic contractility during mouse preimplantation development
Source: PLoS Biol. 2022 Mar 24;20(3):e3001593. doi: 10.1371/journal.pbio.3001593 (PMC8982894; doi:10.1371/journal.pbio.3001593)
Supplement: Attachment — Submitted filename: 220215ResponseToReferees.docx [file pbio.3001593.s030.docx]

We thank both reviewers once again for their support and constructive criticism as we believe they helped us significantly improve our manuscript.

Reviewers' comments

Rev. 1:

As stated in my previous review for Review Commons, the manuscript has three major conclusions: (i) it provides a quantitative description of oscillatory cortical contraction waves in early mouse embryos; (ii) it shows using a series of perturbation and micromanipulation experiments that frequency and amplitude of these contraction waves is independent of cell size; (iii) it provides correlative evidence and a couple of perturbation experiments suggesting that progressive softening of the oocyte cortex is the cause of the observed 'awakening' of contractions as cleavage divisions proceed.

My major criticism was the lack of direct evidence for a causal relationship between cortical tension and contraction waves. This criticism was partially addressed by the authors in the revised manuscript by combining the perturbation experiment (low-dose latrunculin treatment) with measurement of the cortical tension, and by including an additional perturbation (overexpression of fmnl3). With these additions, I think the manuscript is very well suited for publication in PLoS Biology as a Short Report, as I consider the findings still somewhat preliminary, but certainly of high relevance, which is the exact purpose of the Short Reports, as I understand.

I only have a few minor comments to improve readability:

1. As I explained in my previous review, I do think it is very important to establish that PIV is a valid approach to report on cortical contractions. Therefore, I would suggest moving the panels now shown on FS4 into ideally Figure 1 or into one of the other main figures.

Although we appreciate the point of the referee, we do not think that adding the technical panels of FS4 to F1, which does not use this technique, would be helpful. It would be more relevant to add FS4 to F3, which includes the same kind of analysis. However, F3 is already very large and we do not think the added value of FS4 is sufficient to justify this modification.

2. I would suggest to include the data on FS6 in the last main figure. I think this is a very a nice experiment!

Due to editorial space constraints, we have now merged former F4 and F5. We agree that the data in FS6 would be relevant to former F5 but in our opinion this would make the new figure too large.

3. I would suggest removing FS5 panel H, because without detailed validation these stainings are not easily interpretable.

We agree with the reviewer and we have removed the MYH9, ARP2 and CTTN staining. We kept the FMNL3 staining as we believe that immunostaining of FMNL3 on representative zygote, 2-, 4- and 8-cell stage embryos are relevant to our study and that immunostaining of FMNL3 overexpressing embryos shown in FS6 partially validate the antibody specificity.

Rev. 2:

The authors responded to all my comments by arguing or adding experiments, and the result is that the article is strengthened (both by adding controls, but also mechanistic like the part on Fmnl3).

One key question remains: what is the physiological role of these contractions ? In other word, what is the long-term effect of disrupting the appearance/characteristics of PeCoWaCo on the subsequent development of the embryo? This question could be addressed by tuning the intensity of PeCoWaCo in the zygote or at the 2-cell stage, and monitoring the development of the embryo up to the blastocyst stage to assess the outcome on embryo development. This question was only partly addressed by Fmnl3 overexpression, as the effect is transient: the
embryos show PeCoWaCo at the end of the 4-cell stage and develop to blastocyst normally. However, since this manuscript is considered a Short Report, this is not necessary and will pave the way for future research.

Minor:
- The scale bar values are still missing in the legends for Figures 2B, 2F, 4A, 4D 5A.

We apologize for this and thank the reviewer for bringing this to our attention. We have added the missing scale bar values in the legends.

- The tables legends are wrong sometimes (Table 9 is related to figure S5 and not 5, Table 8 is related to figure 5 and not S5).

We apologize for this mistake and thank the reviewer for bringing this to our attention. We corrected this mismatch between the tables and their legends. As we have merged F4 and F5, we also merged table 7 and 8 together and therefore table 9 became table 8.
